# The impact of bolus on clinical outcomes for post-mastectomy breast cancer patients treated with IMRT: data from China

**DOI:** 10.1186/s13014-024-02456-z

**Published:** 2024-05-28

**Authors:** Tao Jiang, Jiao Tian, Peijie Lei, Chunliu Meng, Jialei Fu, Lianjing Cao, Jingjing Cheng, Fei Zhou, Hongjun Zhang, Hao Song, Haijun Lu, Xiaojuan Wei

**Affiliations:** 1 Department of Radiation Oncology, The Affiliated Hospital of Qingdao University, Qingdao, 266700 China; 2 Department of Medicine, Qingdao University, Qingdao, 266700 China

**Keywords:** Breast cancer, Intensity-modulated radiotherapy, Bolus, Local recurrence, Survival

## Abstract

**Purpose:**

This study aims to investigate the effects of chest wall bolus in intensity-modulated radiotherapy (IMRT) technology on clinical outcomes for post-mastectomy breast cancer patients.

**Materials and methods:**

This retrospective study included patients with invasive carcinoma ((y)pT0-4, (y)pN0-3) who received photon IMRT after mastectomy at the Affiliated Hospital of Qingdao University from 2014 to 2019. The patients were divided into two groups based on whether they received daily bolus application or not, and the baseline characteristics were matched using propensity score matching (PSM). Cumulative incidence (CI) of local recurrence (LR), locoregional recurrence (LRR), overall survival (OS) and disease-free survival (DFS) were evaluated with a log-rank test. Acute skin toxicity and late radiation pneumonia was analyzed using chi-square test.

**Results:**

A total of 529 patients were included in this study, among whom 254 (48%) patients received bolus application. The median follow-up time was 60 months. After matching, 175 well-paired patients were selected. The adjusted 5-year outcomes (95% confidence interval) in patients treated with and without bolus were, respectively: CI of LR 2.42% (0.04–4.74) versus 2.38% (0.05–4.65), CI of LRR 2.42% (0.04–4.74) versus 3.59% (0.73–6.37), DFS 88.12% (83.35–93.18) versus 84.69% (79.42–90.30), OS 94.21% (90.79–97.76) versus 95.86% (92.91–98.91). No correlation between bolus application and skin toxicity (*P* = 0.555) and late pneumonia (*P* = 0.333) was observed.

**Conclusions:**

The study revealed a low recurrence rate using IMRT technology. The daily used 5 mm chest wall bolus was not associated with improved clinical outcomes.

**Supplementary Information:**

The online version contains supplementary material available at 10.1186/s13014-024-02456-z.

## Introduction

Studies have shown that post-mastectomy radiation therapy (PMRT) can improve the local control and overall survival for breast cancer patients with advanced disease or certain high-risk pathologic features [1, 2]. Chest wall (including the residual breast glandular tissue, skin and subcutaneous lymphatic plexus) and regional lymphatics have been identified as the most frequent sites of recurrences after PMRT [3, 4]. To prevent recurrence, it is crucial to ensure that the chest wall can receive an adequate radiation dose during PMRT. During the radiotherapy process, high-energy X-ray beams can only reach their maximum radiation dose after penetrating a certain depth into the human tissue, known as the build-up effect or skin sparing effect. Ryan Manger et al. reported that the superficial dose ranged from 40 to 72% of prescription dose without bolus, and increased to 85–109% of prescription dose with 5-mm tissue-equivalent bolus in photon tangent plan delivered to a thorax phantom [5].Therefore, it may be necessary to place a tissue-equivalent bolus on skin's surface to enhance the surface dose of chest wall [6].

However, until now, there has not been a clear standard for whether bolus should be used or not. A worldwide e-mail survey revealed that 82% of Americans and 65% of Australasians were inclined to always use a bolus when delivering PMRT. Europeans only showed a higher tendency to use a bolus for specific indications, such as T4 or inflammatory breast cancer [7]. Meanwhile, the thickness and frequency of bolus use also varied significantly between centers and were closely linked to the incidence and severity of radiation dermatitis [8–10].

It is worth noting that previous surveys and studies predominantly examined bolus application in conventional radiotherapy or three-dimensional conformal radiotherapy (3D-CRT), rather than intensity-modulated radiotherapy (IMRT) which imposes stricter constraints on heart or lung dose volume and reduces acute toxicity of PMRT. Hence, there is currently a gap in research exploring the impact of bolus application when utilizing IMRT technology. In addition, for Chinese breast cancer patients, the breast conditions (such as size, thickness) may differ from other regions. Thus, it was supposed that bolus may play a unique role in PMRT for Chinese patients.

So, in this study, we conducted a retrospective study to analyze the clinical outcomes of Chinese breast cancer patients who underwent mastectomy and IMRT, with or without bolus, focusing on local control, skin toxicity, and survival rates. Additionally, we also evaluated the dosimetry characteristics of organs at risk (OAR), particularly the lung and heart.

## Methods and materials

### Study design and patient selection

This study was approved by the Ethics Board of the Affiliated Hospital of Qingdao University. Patients diagnosed with breast cancer between January 1, 2014, and December 31, 2019, who were treated with unilateral mastectomy and either local (chest wall) or locoregional (chest wall and regional nodal) radiation therapy were included. The inclusion criteria in this study were: (1) a diagnosis of breast cancer confirmed from pathological specimens; (2) patients could be treated with neoadjuvant chemotherapy (ypT0-4, N0-3) or adjuvant chemotherapy (pT1-4, pN0-3); (3) received modified radical mastectomy and routine level I/II axillary lymph node dissection; (4) received post-mastectomy IMRT. Meanwhile, the exclusion criteria were: (1) with M1 disease; (2) received breast reconstruction; (3) treated with 2D technology or 3D-CRT; (4) received radiation therapy after chest wall recurrence; (5) treatment termination due to personal or financial reasons. The decision of bolus application was selectively made for high-risk patients, based on the professional judgment of our oncologists. Patients with skin involvement applied bolus, which was a consensus among us oncologists. The other high-risk factors were collected through a survey conducted prior to our analysis. The factors that influenced oncologists to choose bolus were listed in Table 1 for further analysis using PSM.

**Table 1:** Patient baseline characteristics

Characteristics	Before PSM		*P* value	After PSM		*P* value
	No-bolus (N = 275) No. of patients (%)	Bolus (N = 254) No. of patients (%)		No-bolus (N = 175) No. of patients (%)	Bolus (N = 175) No. of patients (%)	
Age, median (range)	54 (33–83)	55 (32–82)	0.991	53 (31–79)	54 (33–78)	0.841
(y) pT stage			0.230			0.429
0–2	264 (96.00%)	238 (93.70%)		169 (96.57%)	166 (94.86%)	
3–4	11 (4.00%)	16 (6.30%)		6 (3.43%)	9 (5.14%)	
(y) pN stage			0.121			0.587
0–1	155 (56.36%)	160 (62.99%)		105 (60.00%)	100 (57.14%)	
2–3	120 (43.64%)	94 (37.01%)		70 (40.00%)	75 (42.86%)	
Subtype			0.007*			0.062
Luminal A	44 (16.00%)	47 (18.50%)		21 (12.00%)	37 (21.14%)	
Luminal B	171 (62.20%)	124 (48.80%)		111 (63.43%)	92 (52.57%)	
Triple negative	32 (11.60%)	44 (17.30%)		27 (13.43%)	24 (13.71%)	
Her-2 positive	24 (8.70%)	38 (15.00%)		16 (11.14%)	22 (12.58%)	
Unknown	4 (1.50%)	1 (0.40%)				
Grade			0.914			0.912
1–2	162 (58.90%)	150 (59.05%)		108 (61.71%)	109 (62.28%)	
3	91 (33.10%)	86 (33.86%)		67 (38.29%)	66 (37.72%)	
Unknown	22 (8.00%)	18 (7.09%)				
Hormone therapy			0.200			0.560
Yes	193 (70.18%)	165 (64.96%)		120 (68.57%)	125 (71.43%)	
No	82 (29.82%)	89 (35.04%)		55 (31.43%)	50 (28.57%)	
Peri operational chemotherapy			0.001*			0.722
Adjuvant	197 (71.64%)	144 (56.69%)		127 (72.57%)	124 (70.86%)	
Neoadjuvant	66 (24.00%)	100 (39.37%)		48 (27.43%)	51 (29.14%)	
No chemotherapy	12 (4.36%)	10 (3.94%)		0 (0.00%)	0 (0.00%)	
Anti-Her2 therapy			0.150			0.726
No	208 (75.60%)	178 (70.10%)		121 (69.14%)	124 (70.86%)	
Yes	67 (24.40%)	76 (29.90%)		54 (30.86%)	51 (29.14%)	
Margin			0.788			0.792
Negative	253 (50.50%)	226 (45.10%)		168 (96.00%)	167 (95.43%)	
Positive	9 (1.80%)	11 (2.20%)		7 (4.00%)	8 (4.57%)	
Close	1 (0.20%)	1 (0.20%)				
LVI			0.127			0.693
Positive	122 (44.36%)	93 (36.61%)		65 (37.14%)	72 (41.14%)	
Negative	83 (30.18%)	79 (31.10%)		58 (33.14%)	57 (32.57%)	
Unknown	70(25.46%)	82(32.29%)		52(29.72%)	46(26.29%)	

ER: estrogen receptor, PR: progesterone receptor, Her2: human epidermal growth factor receptor 2, LVI: lympho-vascular invasion, IQR: inter quartile range, PSM: propensity score matching

**Fig. 1:**
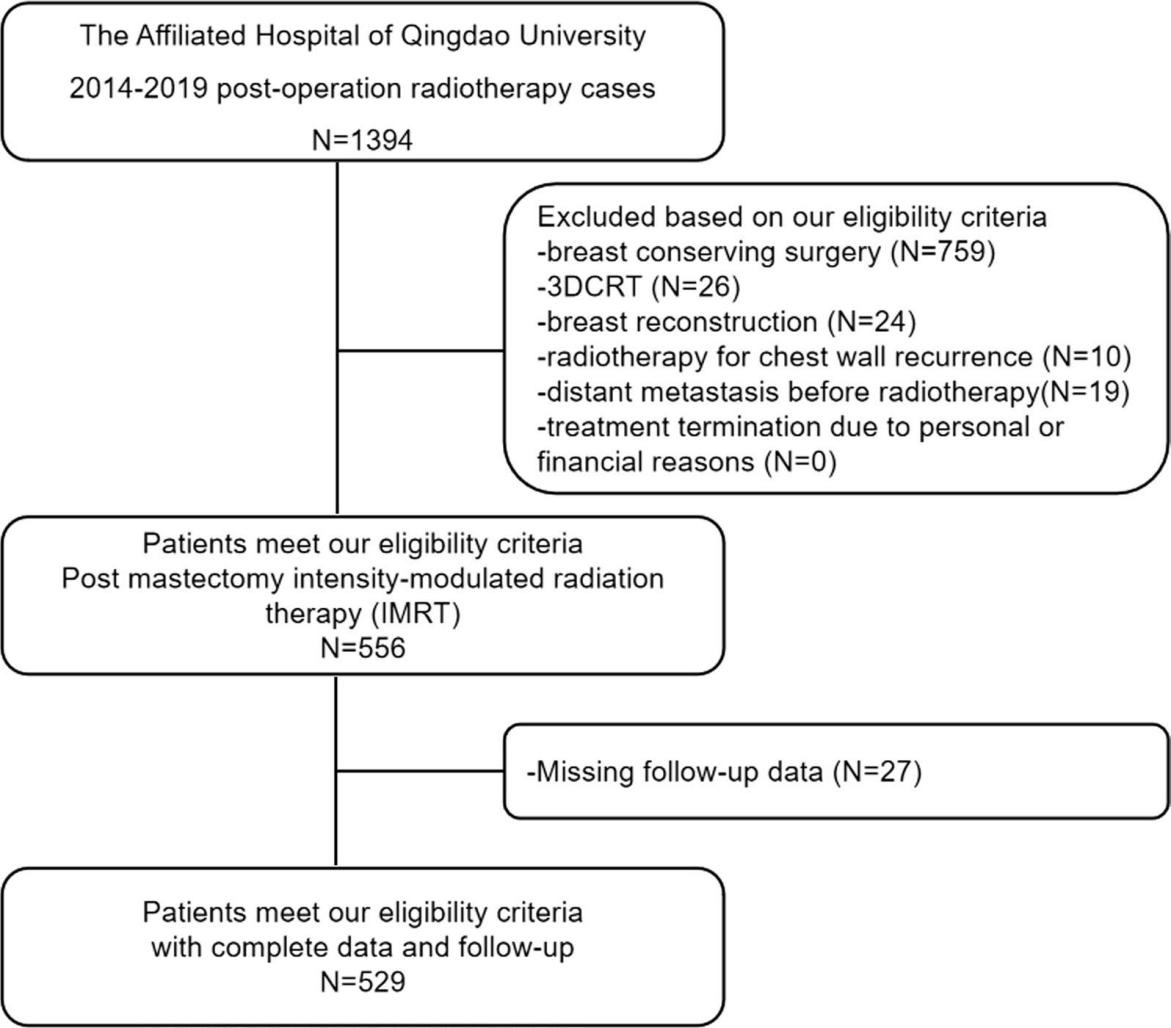
Consort diagram showing the flowchart of this study. 3DCRT: three-dimensional conformal radiation therapy, IMRT: intensity modulated radiotherapy, PSM: propensity score matching

The detailed patients selecting process is shown in Fig. 1.

### Surgery and systematic therapy

All patients recruited for this study underwent a modified radical mastectomy and received routine level I/II axillary lymph node dissection. According to standard treatment guidelines, patients with node-positive breast cancer are typically given an anthracycline-containing regimen followed by taxane administration as perioperative systemic therapy. Patients with positive hormone receptors were given adjuvant hormone therapy, with tamoxifen or aromatase inhibitors being administered based on menopausal status. Patients with HER2 overexpression were treated with trastuzumab.

### Radiation therapy and bolus application

The majority of our patients (98.68%) utilized the filed-in-filed (FIF) technique, while a small percentage (1.32%) applied volumetric modulated arc therapy (VMAT). All patients received a prescription dose of 5000 cGy, divided into 25 fractions, using 6MV X-ray radiation. Patients with node-positive disease underwent locoregional radiation therapy following mastectomy. Contouring of the clinical target volume (CTV) was based on the Radiation Therapy Oncology Group (RTOG) guidelines. The CTV was expanded by 2–5 mm non-uniformly to create the planning target volume (PTV). In cases where the primary tumor is located in the central or medial part of the breast, radiotherapy of internal mammary nodes was considered. Treatment planning for IMRT plans were performed on the Eclipse treatment planning system (TPS) version (Varian Medical Systems, Palo Alto, CA, USA). Each IMRT plan was optimized using the photon Optimization Method (PO), and the resulting dose distribution was evaluated using the Anisotropy Analysis Algorithm (AAA) with a grid square resolution of 2.5 mm. The plan involved employing 5 to 8 radiation fields, with the collimator bar rotation angle ranging from 0° to 30°. The optimization process took into account the uniformity and dose coverage of the PTV. All plans achieved clinically acceptable coverage of the modified PTV (mPTV), with mD95% of the target area received a prescription dose. Furthermore, all risk factors met the clinical goals. Planning approach was similar for both groups. These planning qualities have been estimated according to the ‘RATING’ score sheet [11].

**Fig. 2:**
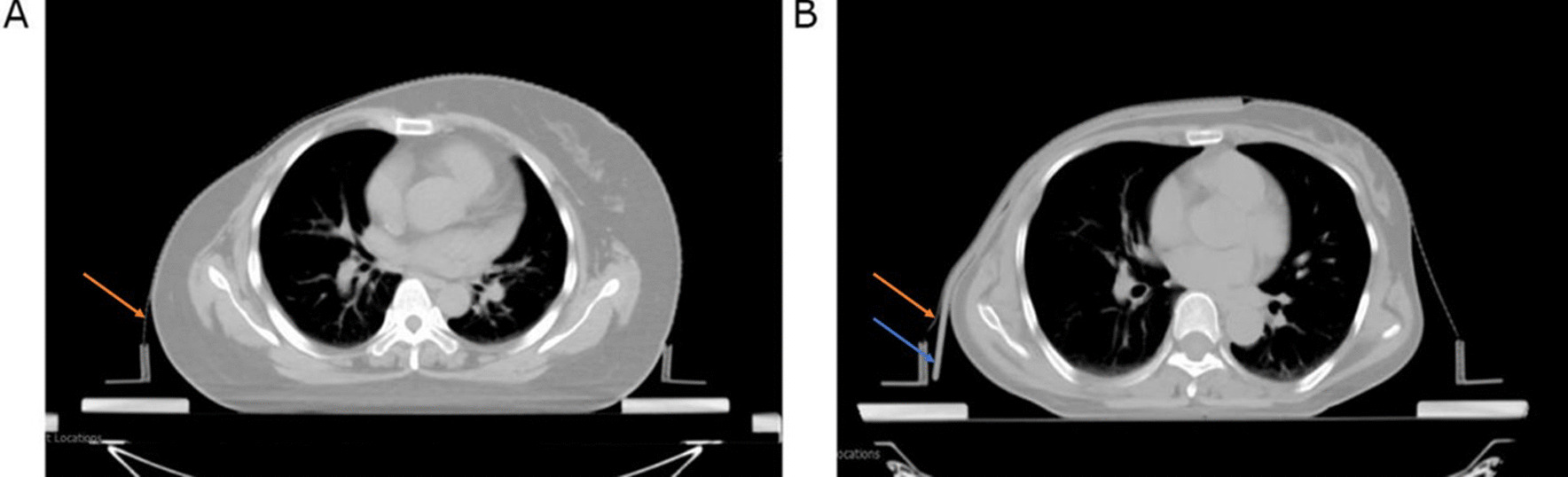
CT scan images with and without bolus application. **A** Thermoplastic shell was used to immobilize the patient's position during the scan; **B** Bolus was applied between the thermoplastic shell and chest wall.  : thermoplastic shell;  : bolus between the thermoplastic shell and chest wall

During the radiotherapy process, patients were positioned supine on a breast board with both arms abducted above their head. To immobilize their chests, a thermoplastic shell (YC-M10-501AHW, Shenzhen Teng Unisoft Technology CO., LTD) extending from the supraclavicular fossa to the bottom of the ribcage was used [12] (Fig. 2A). For patients with high-risk factors selected by oncologists, a 5 mm bolus was placed daily between the chest wall and thermoplastic sheet (Fig. 2B). No patients received a chest wall boost.

### Outcome measures and data collection

The primary endpoints were local recurrence (LR) and locoregional recurrence (LRR), while the secondary outcomes were disease free survival (DFS), overall survival (OS), and radiation related adverse events, including acute skin toxicity and late radiation pneumonia. LR was defined as recurrence within the ipsilateral skin and/or subcutaneous tissue, while LRR was defined as recurrence within the ipsilateral skin and/or subcutaneous tissue and the regional nodes. The term 'relapse' was used to describe the initial occurrence of either locoregional or distant tumor metastasis. DFS was calculated as the time from the date of diagnosis to the occurrence of tumor relapse. OS was defined as survival time from time of diagnosis. All of the patients were followed until January 31, 2023.The follow-up methods included outpatient reexamination, telephone follow-up, and medical record review.

The occurring time of skin side effect including dry or moist desquamation and the degree of erythema were assessed. Based on the RTOG classification [13], the primary distinction between grades 2 and 3 was the presence of moist desquamation and tenderness, whereas grade 4 was characterized by necrosis, ulceration, or bleeding. During the treatment of our patients, we regularly inspected their chest wall and recorded observations of their skin condition. For statistical analysis, we collected the most severe skin toxicity recorded during radiotherapy. During the follow-up phone calls, patients were asked to describe their skin condition, with a specific focus on keywords such as dry peeling, edema, wet peeling, or ulcer. These descriptions, along with the medical records, were evaluated by two doctors simultaneously to ensure accurate assessment. Treatment interruption and early termination due to side effects and other reasons were collected through the medical records and the treatment scheduling system, confirmed by follow-up phone calls.

The severity of radiation pneumonia was assessed based on the Radiation Therapy Oncology Group (RTOG) criteria. Heart and lung dosimetry were calculated using the treatment planning system (TPS). The chest CT films or X-rays of the patients were compared before and after radiotherapy to determine the presence of radiation-induced lung injury.

### Data analysis and statistical considerations

To minimize potential selection bias and confounders, we used propensity score matching (PSM) to control for differences in baseline characteristics. Patients in the entire cohort were matched at a 1:1 ratio using a caliper of width equal to 0.2 without replacement, simulating random allocation using SPSS. Clinicopathological characteristics and treatments data between two groups (bolus versus no-bolus) were compared using Pearson’s chi-square tests. Cumulative incidence was used to evaluate LR and LRR, and Kaplan–Meier survival was applied to analyze breast cancer DFS and OS. Additionally, Pearson’s chi-square was used to analyze skin toxicity and late radiation pneumonia, while the independent t-test was used to analyze the differences in OAR dosimetry. All tests were considered two-sided, and a statistically significant *P*-value was determined to be less than 0.05. The statistical analyses were performed using SPSS (version 27).

## Results

### Patient characteristics

This study included a total of 529 patients, with 254 (48%) patients in the bolus group and 275 (52%) patients in the no-bolus group. The median follow-up period was 60 months, ranging from 11 to 102 months. There were significant differences in patient characteristics between the bolus group and the no-bolus group, in terms of pathological subtype (*P* = 0.007) and whether they received perioperative chemotherapy or not (*P* = 0.001).After applying a 1:1 ratio of PSM, a total of 175 paired patients were selected, effectively minimizing potential selection bias. The patients' characteristics were displayed in Table 1.

Out of the 529 patients from whom clinical outcomes were collected, 16 patients (16/529, 3%) experienced interruptions or early terminations. 9 patients of them were unable to complete the full treatment period (mean fractions: 22.67, range: 16–24) due to chest wall skin ulceration and bleeding. 7 patients had treatment interruptions due to skin toxicity but managed to finish all 25 fractions (mean interruption days: 7.33, range 4–10). Furthermore, the medical records indicated that 1 patient had a 5-day suspension due to diarrhea. No correlation was shown between bolus application and treatment interruption or early termination (Table 1 in Supplementary material). The mean radiation dose delivered was 49.92 Gy (range, 32–50 Gy). The duration of treatment was on average 33.93 days (range, 22–45 days).

### Prognosis roles for bolus application

**Fig. 3:**
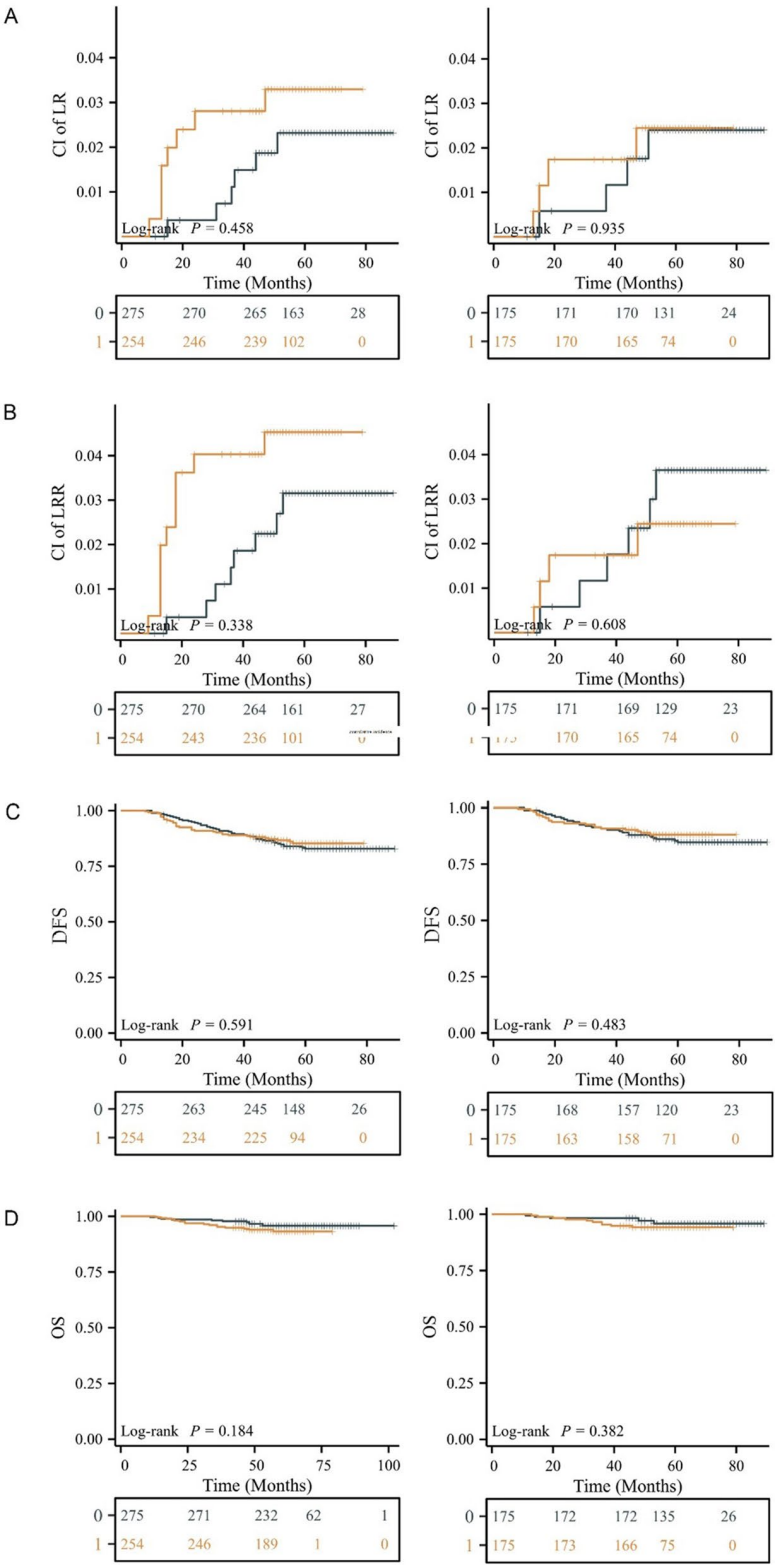
Cumulative incidence (CI) curves comparing LR (**A**) and LRR (**B**), Kaplan Meier curves comparing DFS (**C**) and OS (**D**) between bolus group and no bolus group. **A** CI of LR before PSM (left side), CI of LR after PSM (right side); **B** CI of LRR before PSM (left side), CI of LRR after PSM (right side); **C** K-M curves of DFS before PSM (left side), K-M curves of DFS after PSM (right side); **D** K-M curves of OS before PSM (left side), K-M curves of OS after PSM (right side).  : bolus group;  : no bolus group. LR: local recurrence, LRR: locoregional recurrence, DFS: disease free survival, OS: overall survival, CI: cumulative incidence, K-M: Kaplan Meier, PSM: propensity score matching

The cumulative incidence (CI) of LR and LRR, and the survival outcomes were shown in Fig. 3. The adjusted 5-year outcomes (95% confidence interval) in patients treated with and without bolus were, respectively: CI of LR 2.42% (0.04–4.74) versus 2.38% (0.05–4.65) (Fig. 3A), CI of LRR 2.42% (0.04–4.74) versus 3.59% (0.73–6.37) (Fig. 3B), DFS 88.12% (83.35–93.18) versus 84.69% (79.42–90.30) (Fig. 3C), OS 94.21% (90.79–97.76) versus 95.86% (92.91–98.91) (Fig. 3D). There was no significant difference between two groups in terms of clinical outcomes in both the entire cohort (CI of LR: *P* = 0.458, CI of LRR: *P* = 0.338, DFS: *P* = 0.591, OS: *P* = 0.184) and PSM matched cohort (CI of LR: *P* = 0.935, CI of LRR: *P* = 0.608, DFS: *P* = 0.483, OS: *P* = 0.382), as shown in Fig. 3. Among the 529 patients, 4 cases were diagnosed with pathological skin invasion and ulcer formation (T4b). Bolus was applied in all these 4 cases, and no instances LR or distant metastasis were observed during the follow-up period.

### Radiation related adverse events

Table 2 displays the acute skin toxicity and late radiation pneumonia in the entire cohort and PSM matched cohort. Both bolus and no bolus groups showed rare instances of Grade 3–4 toxicity and late radiation pneumonia. In the matched cohort, 14 (8%) experienced Grade 3–4 skin toxicity in bolus group, compared to 9 (5.14%) patients treated without a bolus. Grade 1–2 toxicity was observed in 148 (84.57%) patients treated with bolus, while 152 (86.86%) patients in the no bolus group. The Chi-Squared test revealed no significant difference between the two groups (*P* = 0.555). We also investigated the incidence of late radiation pneumonia in patients who applied bolus versus those who did not. Results showed that 6.29% of patients who applied bolus developed late radiation pneumonia, compared to 4% of patients who did not in the PSM matched cohort. All cases of pneumonia were classified as grade 0–2 according to the RTOG/EORTC late radiation morbidity scoring scheme [13], and there was no significant difference between two groups (*P* = 0.333) (Table 2).

**Table 2:** Adverse events in bolus group and no bolus group

Characteristics	Before PSM		*P* value	After PSM		*P* value
	No-bolus (N = 275) No. of patients (%)	Bolus (N = 254) No. of patients (%)		No-bolus (N = 175) No. of patients (%)	Bolus (N = 175) No. of patients (%)	
Acute skin toxicity, n (%)			0.936			0.555
Grade 1–2	239 (86.9%)	218 (85.83%)		152 (86.86%)	148 (84.57%)	
Grade 3–4	16 (5.82%)	16 (6.3%)		9 (5.14%)	14 (8.00%)	
Unknown	20 (7.28%)	20 (7.87%)		14 (8.00%)	13 (7.43%)	
Late pneumonia, n (%)			0.329			0.333
No	262 (95.27%)	237 (93.30%)		168 (96.00%)	164 (93.71%)	
Yes (grade 1–2)	13 (4.73%)	17 (6.70%)		7 (4.00%)	11 (6.29%)	

PSM: propensity score matching

### Dosimetric differences of heart and lung according to bolus application

Table 3 presents a comparison of dosimetric differences between Bolus+ and Bolus− for post-mastectomy IMRT treatment of left and right breast cancer. Bolus group showed significant lower Dmean (*P* < 0.001), V20% (*P* = 0.002), V30% (*P* = 0.005) of the ipsilateral lung and lower Dmean (*P* = 0.01) of the heart in patients with left breast cancer in cohort after PSM compared to plans without bolus. Similarly, for right breast cancer patients, plans including bolus showed a lower mean radiation dose to the right lung compared to plans without bolus (*P* = 0.0297) in cohort after PSM.

**Table 3:** Comparison of dosimetry parameters of lung and heart between bolus group and no bolus group in left breast cancer and right breast cancer

Characteristics (Left breast cancer)	Before PSM		*P* value	After PSM		*P* value
	No-bolus (N = 150)	Bolus (N = 132)		No-bolus (N = 97)	Bolus (N = 91)	
Dmean of ipsilateral lung, median (IQR)	1525.2 (1427.1, 1697.9)	1463.2 (1357.2, 1542.6)	< 0.001*	1569.9 (1435.8, 1726.6)	1466.9 (1369.5, 1544)	< 0.001*
V20% of ipsilateral lung, median (IQR)	24.5 (21.618, 26.995)	22.85 (20.9, 24.625)	< 0.001*	24.6 (21.69, 27.2)	22.9 (20.995, 24.65)	0.002*
V30% of ipsilateral lung, median (IQR)	16 (14.33, 18)	15.15 (13.2, 16.5)	0.001*	16 (14.7, 18.23)	15.1 (13.495, 16.52)	0.005*
Dmean of heart, mean ± sd	1203.2 ± 360.39	1090.1 ± 300.83	0.004*	1250.1 ± 357.41	1126.1 ± 285.41	0.010*
V30% of heart, median (IQR)	6.16 (3.48, 9.075)	5.45 (3.7825, 8.05)	0.213	6.5 (3.6, 9.47)	5.95 (4.2, 8.3)	0.432

Characteristics (Right breast cancer)	Before PSM		*P* value	After PSM		*P* value
	No-bolus (N = 125)	Bolus (N = 122)		No-bolus (N = 78)	Bolus (N = 84)	
Dmean of ipsilateral lung, median (IQR)	1500.1 (1436, 1657.7)	1479.2 (1371.9, 1604.6)	0.022*	1530.8 (1450.4, 1672.6)	1481.3 (1375.2, 1614.2)	0.030*
V20% of ipsilateral lung, median (IQR)	23.6 (21.6, 26.49)	23.7 (22, 25.875)	0.994	23.7 (21.45, 27.45)	23.7 (22, 25.575)	0.632
V30% of ipsilateral lung, median (IQR)	15.5 (14, 17.9)	15.2 (13.617, 17.1)	0.251	15.45 (14.025, 17.788)	14.8 (13.4, 17.025)	0.193
Dmean of heart, median (IQR)	536.4 (423.6, 649.1)	508.35 (397.07, 619.52)	0.130	544.25 (450.55, 664.93)	518.15 (390.8, 625.07)	0.067

## Discussion

In the current study, we evaluated the effect of bolus on patients treated with mastectomy and IMRT from China. Our study is the first to report on the clinical outcomes of breast cancer patients who received IMRT technology following mastectomy.

Through a questionnaire survey, we identified the factors that impact the application of bolus. In our center, it is widely agreed that patients with skin invasion should receive bolus, aligning with “A Delphi study and International Consensus Recommendations” reported by Orit et al. in 2021 [7]. We identified other high-risk factors that influence doctors' decision to choose bolus and conducted PSM matching. This method aimed to minimize the selection bias between the two patient groups.

After matching patients' baseline characteristics, the 5-year LR, LRR, DFS and OS were 2.42%, 2.42%, 88.12% and 94.21%, respectively, and, there was no significant difference in acute skin toxicity (*P* = 0.555) and late radiation pneumonia (*P* = 0.333). Meanwhile, the use of bolus had advantages in reducing the exposure dose to the lungs and heart. The use of bolus for breast cancer patients undergoing PMRT has been a topic of debate, as evidenced by studies [14–16]and clinician surveys. Typically, LR and LRR were the endpoints of interest. In studies where bolus was routinely used, LRR rates ranged from 6 to 10% [17, 18]. In our study, recurrence rate was lower in the bolus group compared with previously published data [9, 19, 20]. Dahn et al. reported that LR from thirteen studies (n = 3756) were 3.5% with bolus and 3.6% without bolus [9]. The majority of these 13 studies utilized 3DCRT technology. In our study, both the bolus group (2.42%) and the no bolus group (2.38%) exhibited lower rates of LR over a 5-year period. This may be related to the following reasons. Firstly, the treatment modalities in other studies were mainly conventional radiotherapy and 3D-CRT. While, in our study, we used IMRT technique for postoperative radiotherapy. It has been reported that the more fields tangent to the outer contour curve of the chest wall, the more helpful to improve the skin surface dose [5]. Compared with conventional radiotherapy and 3D-CRT, IMRT involves a greater number of irradiation angles, which increases the probability that the field will be tangential to the mammary gland contour, allowing a wider range of skin surface doses to be enhanced. Secondly, in our study, most of patients in our study (513/529, 96.98%) completed treatment as planned. Pignol et al. conducted a prospective series to assess acute toxicity in patients receiving PMRT. They found that as the thickness and frequency of bolus application increased, pain and grade 3 moist desquamation also increased significantly [14]. In our study, we also observed a higher incidence of grade 3–4 skin reactions in the bolus group compared to the no bolus group (8% versus 5.14%), suggesting that the daily use of bolus may contribute to the increased severity of skin toxicity. Dahn et al. reviewed 27 studies and reported that the use of bolus led to higher rates of acute grade 3 radiation dermatitis (9.6% with bolus). Our incidence of grade 3–4 skin reactions (8% with bolus) was a bit lower than the review reported [9]. Tieu et al. reported daily 1-cm whole chest wall bolus may increase the risk of chest wall recurrence by increasing the risk of acute skin reactions and hence early cessation of the radiotherapy course [19]. These studies were crucial for our clinical practice in understanding the impact of bolus-induced acute skin reactions. During the treatment period, patients who experienced ulcer on their chest wall were recommended to sterilize the injured skin with iodophor, apply growth factor ointment and betamethasone cream, and then cover it with gauze every day. Early intervention in managing side effects likely contributed to the successful completion of treatment by the majority of our patients.

Our findings indicated that the application of chest wall bolus did not lead to a decrease in the LR (2.42% versus 2.38%). This observation is consistent with the conclusion published by Nichol et al. in 2021[10]. Lung and heart injuries were also of concern for breast postoperative radiotherapy. Our findings indicated that the bolus group exhibited lower dosimetry in the lungs and heart on the ipsilateral side with similar treatment plan parameters, the addition of the chest wall bolus causes a forward movement of the isodose line, which ultimately leads to a decrease in the irradiation dose to the ipsilateral heart and lungs. However, when considering our follow-up results of radiation pneumonitis, it appears that the decrease in lung dose does not result in significant clinical benefits.

Notably, the pneumonia images from both groups showed only mild changes. None of the patients displayed symptoms indicative of severe pneumonia, such as severe cough, wheezing, and dyspnea. This may be attributed to the advantages of IMRT technology in accurately distributing doses to targets and protecting organs at risk.

There are several limitations to this analysis. Most importantly, due to its retrospective nature, the assessment of radiation damage may not be particularly accurate. Secondly, this study was based on the experience of a single institution, and the number of patients was limited. Further studies involving larger samples are needed to confirm these findings.

## Conclusion

Breast cancer patients who underwent IMRT after radical mastectomy exhibited a low recurrence rate and a low incidence of toxicity. Our retrospective study findings suggest that the use of bolus application did not result in additional reduction in the recurrence rate. Consequently, the majority of oncologists at our center agree that chest wall bolus should no longer be administered to patients undergoing IMRT, unless they have skin invasion.

### Supplementary Information

Additional file 1

## Data Availability

The data sets supporting the results of this article are included within the article and its figures and tables.

## Abbreviations

PMRT: Post-mastectomy radiation therapy
3D-CRT: Three-dimensional conformal radiotherapy
IMRT: Intensity-modulated radiotherapy
OAR: Organs at risk
PSM: Propensity score matching
FIF: Filed-in-filed
VMAT: Volumetric modulated arc therapy
CTV: Clinical target volume
RTOG: Radiation Therapy Oncology Group
PTV: Planning target volume
TPS: Treatment planning system
LR: Local recurrence
LRR: Locoregional recurrence
DFS: Disease free survival
OS: Overall survival
CI: Cumulative incidence
