# Supplementary material for: The impact of bolus on clinical outcomes for post-mastectomy breast cancer patients treated with IMRT: data from China
Source: Radiat Oncol. 2024 May 28;19:64. doi: 10.1186/s13014-024-02456-z (PMC11134933; doi:10.1186/s13014-024-02456-z)
Supplement: Supplementary file 1 — Additional file 1 [file 13014_2024_2456_MOESM1_ESM.docx]

Supplementary Materials

| **529 patients the study included** | **Bolus** | **No-bolus** | ***P* value** |
| --- | --- | --- | --- |
| Interruption or termination of treatment due to skin toxicity (1 case of interruption due to diarrhea was excluded) | 7 | 8 | 0.92 |
| No interruption occurs | 246 | 267 |  |
| **350 patients after PSM matching** | | | ***P* value** |
| interruption or termination of treatment due to skin toxicity | 5 | 4 | 0.736 |
| No interruption occurs | 170 | 171 |  |

Table 1: Pearson correlation analysis between “interruption or termination of treatment due to skin toxicity” and “bolus application” both in the entire cohort and PSM matching cohort.

**Patient characteristics about treatment discontinuation**

Out of the 529 patients from whom clinical outcomes were collected, 16 patients (16/529, 3%) experienced interruptions or early terminations. Within the group of 16 patients, 9 were unable to complete the full treatment period. Specifically, 4 out of these 9 patients completed 24 fractions, another 4 patients underwent 23 fractions, and 1 patient finished 16 fractions due to chest wall skin ulceration and bleeding. Among these 9 patients, 5 patients used bolus, including the patient who completed only 16 sessions. Among the 16 patients who experienced interruptions or terminations, 7 cases had treatment interruptions but managed to finish all 25 fractions. Among these, 6 cases stopped for 4-10 days due to skin toxicity. Notably, 2 of these cases were part of the bolus group and 4 were from the no-bolus group. Furthermore, the medical records indicated that 1 patient had a 5-day suspension due to diarrhea.

A Pearson correlation analysis were performed on the relationship between “interruption or termination of treatment due to skin toxicity” and “bolus application” (Table 1). The result showed no significant correlation.
